# Fidelity evaluation of the *Water First* intervention for promoting tap water consumption in school-age children

**DOI:** 10.1186/s12889-026-28101-w

**Published:** 2026-06-13

**Authors:** Anjali Gupta, Keejeong Ryu, Yadira Peralta, Valeria Ordonez, Charles E. McCulloch, Laura A. Schmidt, Lorrene D. Ritchie, Anisha I. Patel

**Affiliations:** 1 Stanford University School of Medicine, Stanford, CA USA; 2 Quantitative Sciences Unit, Stanford University School of Medicine, Stanford, CA USA; 3 Center for Research and Teaching in Economics, Department of Applied Economics, Aguascalientes, Mexico; 4 Department of Pediatrics, Stanford University School of Medicine, Palo Alto, CA USA; 5 Department of Epidemiology and Biostatistics, University of California San Francisco, San Francisco, CA USA; 6 Philip R. Lee Institute for Health Policy Studies and Department of Humanities and Social Sciences, University of California San Francisco, San Francisco, CA USA; 7 Nutrition Policy Institute, Division of Agriculture and Natural Resources, University of California, Oakland, CA USA

**Keywords:** Obesity, Intervention fidelity, Public health, Water, Beverages

## Abstract

**Background:**

A cluster-randomized controlled trial examining the impact of the *Water First* water promotion and access program in 18 elementary schools found that the intervention increased water consumption and prevented overweight. This study describes a secondary analysis of implementation fidelity and its association with change in students’ beverage intake and body mass index (BMI) z-score in the intervention arm.

**Methods:**

Researchers observed *Water First* fidelity (visible water promotional material, quality of study-provided water sources and drinking vessels in classrooms, school cafeterias, high-traffic areas) 2 times/week during the intervention period, with data collected via yes/no questions. Responses indicating high fidelity were assigned a value of 1 and low fidelity were assigned 0; these were averaged to generate a summary score for each location (0 to 1). Home-engagement activity completion (%) was calculated per student based on returned class assignments. Anthropometrics and past-week frequency of beverage intake (instrument adapted from the validated Beverage and Snack Questionnaire) were assessed at baseline, 7-months, and 15-months. Mixed-effects linear regression models examined associations of fidelity with the primary (water intake) and secondary (sugar-sweetened beverage [SSB], milk, 100% fruit juice intake, BMI z-score) outcomes. The primary predictor was the fidelity score (0.1-unit increase) by time interaction. Beverage frequencies were log-transformed, and regression coefficients were exponentiated to derive the percent change in outcomes.

**Results:**

Fidelity was highest in the cafeteria (mean: 0.87, SD: 0.06) compared to high-traffic water station areas (0.66, SD: 0.15) and classrooms (0.73, SD: 0.13). Mean home-engagement activity completion rate was 0.41 (SD: 0.33). There were no associations of fidelity with water intake. Each 0.1 unit increase in classroom fidelity was associated with a decreased intake of 100% fruit juice (0.95; Cl: 0.91–0.99). Higher high-traffic area fidelity was associated with decreased intake of SSB (0.94; Cl: 0.91–0.98) and plain milk (0.92; Cl: 0.89–0.96). Higher cafeteria fidelity was associated with 0.027 decrease in BMI z-score (Cl: -0.046, -0.008). No significant associations were observed for home-engagement activity completion.

**Conclusions:**

The intervention was largely implemented with high fidelity, with scores ranging from 0.66 to 0.87. Higher cafeteria fidelity was associated with reductions in BMI z-score, while home-engagement activity completion was not associated with changes in beverage outcomes or BMI z-score.

**Trial registration:**

The trial was preregistered (2017–06-09) at clinicaltrials.gov (NCT03181971).

**Supplementary Information:**

The online version contains supplementary material available at 10.1186/s12889-026-28101-w.

## Introduction

The prevalence of obesity and overweight among children in the United States (U.S.) has increased dramatically over the last several decades, with nearly 20% of children now meeting criteria for obesity and 6% for severe obesity [1, 2]. Children who are non-Hispanic Black or Hispanic, and those living in regions with lower median income are more likely to have obesity or overweight [3], which places them at higher risk for many obesity-associated chronic illnesses including type II diabetes, cardiovascular disease, and hypertension [4–6]. A major contributor to obesity in children is sugar-sweetened beverage (SSB) consumption [7]. Because schools offer continuous contact with children and adolescents, several interventions have sought to increase water intake and reduce SSB intake among children in school settings [8–16].

*Water First* is a school-based randomized controlled trial of a water promotion and access intervention implemented in 2016–2019 among fourth grade classes in nine elementary schools compared to nine control schools in northern California serving low-income students [8, 17]. The intervention involved 1) installation of lead-free water stations in cafeterias and high-traffic common areas, 2) provision of cups/reusable water bottles for students, and 3) a 6-month healthy beverage education campaign [17]. An effectiveness study of the *Water First* intervention has been previously published, utilizing a parallel arm clustered-randomized controlled trial design [8]. This study found that intervention students reported a significantly greater increase in the frequency of water consumed as compared to control students at 7-months; this was sustained at 15-months. At 15 months, intervention students also had a significantly lower increase in overweight prevalence compared to control students.

While schools offer an ideal setting for primary prevention efforts in children and adolescents, there are often significant time and resource constraints present [18], which may impact the degree to which effective interventions are implemented. Lack of fidelity is a primary reason that interventions shown effective in controlled trials fail to yield beneficial outcomes when applied in the real world [19]. Therefore, evaluating intervention fidelity is critical to discerning essential features of the intervention, refining the intervention, and understanding the feasibility of implementation in other settings [20]. However, few interventions targeting health behavior changes have thoroughly studied implementation fidelity [20], and even fewer have considered the influence of implementation fidelity on outcomes [19].

So far, there is very limited research evaluating fidelity for school-based drinking water interventions [21, 22]. In one study, researchers in Germany conducted a long-term process evaluation of a school-based water promotion intervention that was implemented in 32 elementary schools [21]. They assessed teacher and staff acceptability of the intervention, teacher implementation of intervention-provided lessons, and post-intervention period water dispenser utilization. Importantly, however, the study did not relate the fidelity of implementation of the intervention with changes in student beverage intake or body mass index (BMI). Another process evaluation of a family-based water intake and beverage consumption intervention in six European countries found overall low teacher and parent implementation scores. However, there were more favorable intervention effects on beverage choices (plain water, prepacked fruit juice, and soft drinks) in preschoolers whose caregivers and teachers had higher implementation scores than lower scores [22].

The present study examines: 1) implementation fidelity for the *Water First* intervention, and 2) the association of implementation fidelity with changes in student beverage intake and BMI z-score. Elements that were implemented with highest fidelity likely represent those that may be most feasible to implement in elementary school settings. Likewise, elements that contributed most to changes in student outcomes represent those that may yield the greatest benefit for future implementation. In contrast, elements that had lower rates of implementation fidelity could be improved in future studies.

## Methods

### *Water First* intervention

The detailed protocol following CONSORT reporting guidelines for the *Water First* intervention is published elsewhere [8, 17]. Briefly, *Water First* was implemented among students in fourth grade classes in nine elementary schools in 2016–2019, with three schools receiving the intervention each academic year and three serving as controls. Eight additional schools began the study in 2019–2020 (4 intervention, 4 control), but activities were halted as schools closed due to the onset of the COVID-19 pandemic. Thus, these schools were not included in the analysis. Eligible schools for randomization were in northern California, served low-income students (≥ 50% free/reduced-price meal-eligible), did not promote water, and had sufficient fourth grade students (*n* > 65 per school) [8, 17]. After schools were randomized to intervention vs. control, 677 consented students received the intervention among 9 schools and 28 classrooms [8]. Tap water stations (3 per school), with water tested and remediated for lead as needed, were installed in high-traffic areas (e.g., outdoor school playgrounds, common areas) and cafeterias. Cups were also available next to the tap water station in the cafeteria.

*Water First* also provided water promotion materials, including posters and signage in classrooms, cafeterias, and high-traffic areas (Supplemental Figures), and students received a reusable water bottle for use in school and at home. School staff were responsible for putting up posters, putting out cups, and cleaning water dispensers in cafeterias/high traffic areas, and teachers were responsible for classroom posters and filling and cleaning student water bottles. Additionally, students received eight 15-min in-class lessons, four of which were accompanied by short family home-engagement activities on the health, fiscal, and environmental benefits of drinking water. These in-class lessons were delivered by researchers in all study classrooms. *Water First* not only encouraged water intake, but also discouraged the intake of SSBs, promoted unflavored over flavored milk, and encouraged a 1 serving per day limit of 100% juice. Study assessments, including surveys and anthropometric measurements, occurred at baseline, 7-months, and 15-months. Follow-up assessments were completed by 94.4% of participants (*n* = 639) at 7-months and 80.9% (*n* = 548) at 15-months [8].

Signed parental consent and student assent was obtained for study participation. Institutional review boards at the University of California, San Francisco, and Stanford University approved study protocols. The study was preregistered (2017–06–09) at clinicaltrials.gov (NCT03181971) [23]. The present analysis was restricted to the intervention arm of the study.

### Fidelity scores

Implementation fidelity was measured through direct observation by researchers situated in the intervention schools and classrooms, two times per week over a 3–4-month period. Data were collected separately for fidelity within classrooms, cafeterias, and high-traffic area water stations via a series of yes/no questions (Table 1).

**Table 1:** Criteria for fidelity^1^ checks in *Water First* intervention schools

Locations	Program Component	Yes/No Questions^2^
Water Stations in High-Traffic Areas	Promotion	Are the emoji decals up on the station? Are there posters near the station?
	Environment	Are there any other factors that could influence the appeal of the station? (no = higher fidelity)
Water Station in the Cafeteria	Promotion	Are the emojis on the water dispensers? Are there posters present?
	Environment	Are the water dispensers filled? Are cups available? Are there any other factors that could influence the appeal of the dispensers? (no = higher fidelity)
Classroom	Promotion	Are the emoji decals up near the classroom sink? Are the posters up in the classroom?
	Environment	Are there Share the Love, Share the Water bottles on the students’ desks? Does the teacher have the Share the Love, Share the Water bottle on their desk or visible in the classroom? Are there any other factors that could influence the appeal of the water source? (no = higher fidelity)

^1^Fidelity scores were generated by assigning a value of 1 to responses indicating high fidelity, and 0 to responses indicating low fidelity. Responses were averaged to generate a summary fidelity score for each location incorporating all questions

^2^Responses of “yes” indicate higher fidelity unless otherwise noted

In cafeteria checks, researchers noted the availability of filled water dispensers and cups, visibility of promotional material, and any factors that may reduce the appeal of the water source. At high-traffic area water stations, researchers assessed the visibility of promotional material, and any factors that may reduce the appeal of the water source. At the classroom level, researchers assessed teacher and student usage of study-provided water bottles, visibility of water promotional materials (posters, emoji decals), and any factors that may reduce the appeal of the water source (e.g., dirty water basin). Because classroom lessons were delivered by researchers in all study classrooms (100% fidelity), they are not included in this analysis.

Fidelity scores were generated separately for classroom, cafeteria, and water stations (Table 1). We assigned a value of 1 to individual yes/no question responses indicating high fidelity, and 0 to responses indicating low fidelity. We then averaged the responses to generate a summary fidelity score between 0 and 1 for each location. For descriptive analyses, promotion (i.e., posters and signage) and environment (i.e., factors related to water access) sub-scores were created to separately describe implementation fidelity for those components of the intervention.

### Home-engagement score

Students were given four short home-engagement activities to complete with their families. The four activities involved: 1) viewing a video about the benefits of drinking water and drawing their family’s favorite part; 2) calculating the amount of sugar in a sweetened drink at home; 3) creating a family “beverage contract” to commit to healthier habits at home; and 4) collaborating with family to develop a recipe to naturally flavor water as an alternative to sugary drinks. Students turned in completed activities to school teachers, and percent completion of home-engagement activities was calculated per student.

### Outcomes

The primary outcome for this fidelity evaluation was students’ overall water intake as measured at baseline, 7-months, and 15-months using an instrument adapted from the validated Beverage and Snack Questionnaire [24] that assessed past-week frequency of beverage consumption. Briefly, for each beverage item, seven frequency response options were provided, ranging from “never or less than once/week” to “4 + times/day”; participants marked how often in the previous week they consumed each item “at school” and “away from school” [24]. Secondary outcomes were intakes of other beverages (SSB, plain milk, flavored milk, 100% fruit juice) and BMI z-score. Frequency of beverage intake was selected given significant differences observed between intervention and control students in the *Water First* trial [8]. Height and weight of students was measured at all three timepoints using standardized protocols from the National Health and Nutrition Examination Survey (NHANES) [25]. BMI z-score was calculated using Centers for Disease Control and Prevention growth curves. While overweight prevalence was the primary outcome for the trial [8], we chose BMI z-score as this continuous variable was thought to provide more power, which was important given that this study was limited to the intervention arm only. Covariates included self-reported age, gender, and Hispanic ethnicity.

### Statistical analysis

Descriptive statistics (mean and standard deviation [SD] for continuous variables; frequency [%] for categorical variables) were used to categorize students’ demographic characteristics, frequency of beverage intake, and BMI z-score, as well as *Water First* implementation fidelity for the classroom, water station, and cafeteria components of the intervention. Mixed-effects linear regression models, adjusted for students’ ethnicity, gender, and age with random intercepts for student, school, and classroom, examined associations of fidelity with the primary outcome (water intake) and secondary outcomes (SSB, plain milk, flavored milk, 100% juice intake, BMI z-score). Time was analyzed as a continuous variable (coded 1, 2, and 3 for baseline, 7-months, and 15-months, respectively). The primary predictor was the fidelity score (0.1-unit increase) by time interaction. Beverage intake frequencies were log-transformed, and regression coefficients were exponentiated to derive the intervention change in outcomes and to accommodate the skewed nature of the intakes. Thus, for beverage frequency outcomes, estimates of greater than 1 suggest an increase, while those less than 1 suggest a decrease; for BMI z-score, estimates of greater than 0 suggest an increase, while those less than 0 suggest a decrease. Because the main intervention components (e.g., researcher-led lessons, water promotion efforts) were implemented between baseline and 7-months with water access components continuing from 7-months to 15-months, in sensitivity analyses, time was evaluated as a categorical variable to obtain separate estimates for baseline and 7-months vs. 7-months and 15-months. All analyses were conducted in SAS version 9.4, with two-sided significance set at *p* < 0.05; due to the exploratory nature of this analysis, we did not adjust for multiple comparisons.

## Results

Among students in the intervention arm of the *Water First* study (*N* = 677), the mean (SD) age was 9.63 (0.44), 47% were female, and 73% identified as Hispanic (Supplemental Table 1). Intake of water (times/day [SD]) was 6.06 (4.30) at baseline, 7.10 (5.14) at 7-months, and 6.25 (4.71) at 15-months follow-up. Intake of SSB (times/day [SD]) was 2.48 (2.76) at baseline, 1.83 (2.36) at 7-months, and 1.57 (1.84) at 15-months. Intake of milk, flavored milk, and 100% juice are provided in Supplemental Table 1. Mean (SD) BMI z-score was 0.91 (1.13) at baseline, 0.93 (1.10) at 7-months, and 0.93 (1.14) at 15-months. With 677 students, 9 schools, and 28 classrooms included in the analysis, there were 75 students included on average per school and 24 per classroom, with an overall participation rate of 89% among students in included classrooms.

**Fig. 1:**
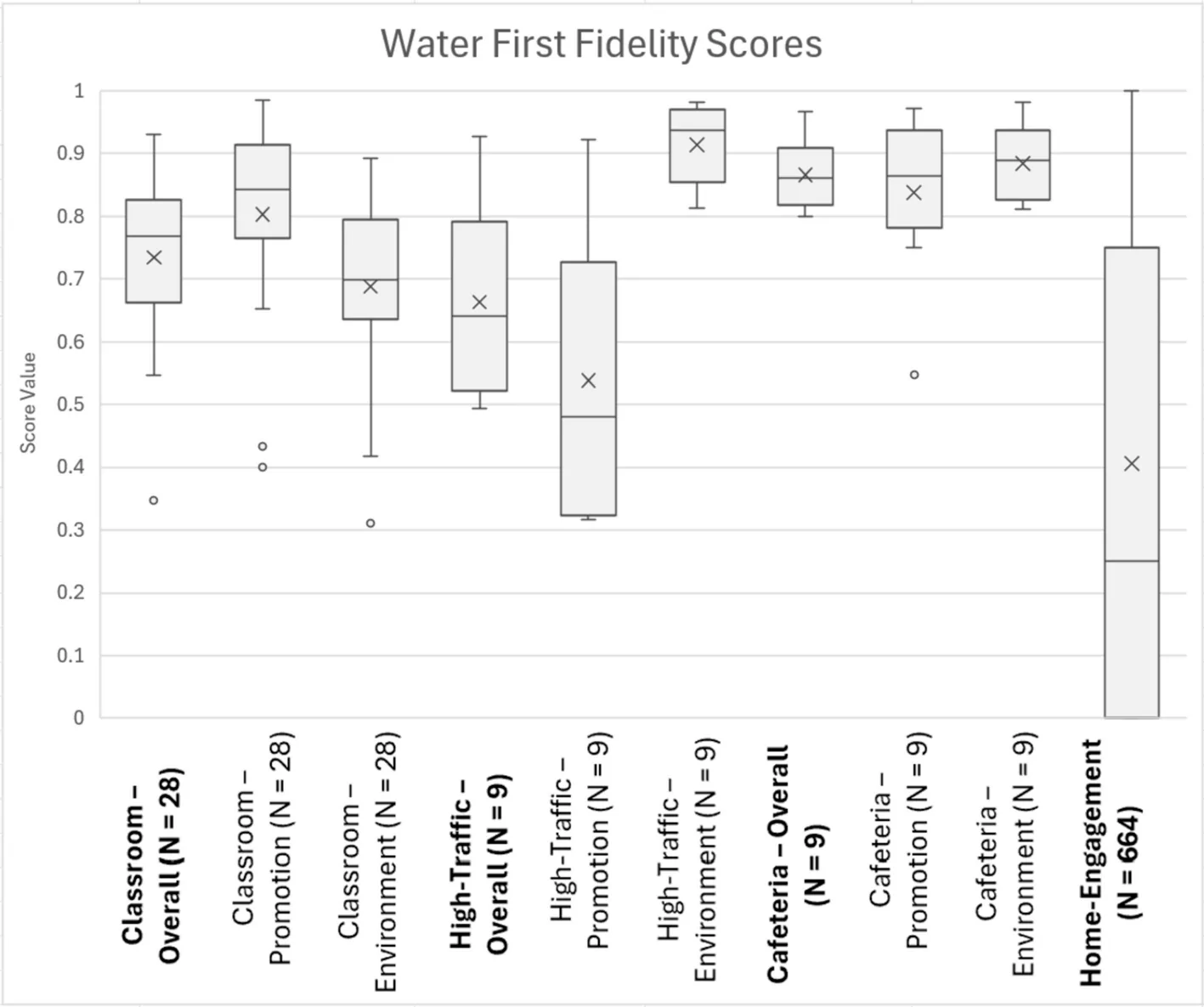
Box-and-whisker plots of *Water First* fidelity scores for classrooms, cafeterias, high-traffic areas, and home-engagement activities. For classrooms (*N* = 28 classrooms), cafeterias (*N* = 9 schools), and high-traffic areas (*N* = 9 schools), separate scores are provided for the water promotion (e.g., posters/signage) and environment (e.g., access to water sources) components of the program; overall score incorporates all components. All scores range from 0 (low fidelity) to 1 (high fidelity)

Descriptive statistics for the fidelity scores and home-engagement score (mean [SD]) are provided in Supplemental Table 2 and Fig. 1. Implementation fidelity was highest in the cafeteria (0.87 [0.06]) compared to high-traffic area water stations (0.66 [0.15]) and classrooms (0.73 [0.13]). For the cafeteria, similar fidelity was noted between the promotion (0.84 [0.13]) and environment (0.88 [0.06]) components of the intervention. For high-traffic area water stations, fidelity was lower for promotion (0.54 [0.22]) compared to environment (0.91 [0.06]). For classrooms, fidelity was higher for promotion (0.80 [0.16]) compared to environment (0.69 [0.14]). Mean (SD) home-engagement activity completion rate was 0.41 (0.33).

In sensitivity analyses with time as a categorical variable, trends were similar to those observed for the overall analysis (Supplemental Tables 3a-b); thus, we present main results incorporating all three timepoints. There were no associations of implementation fidelity and water intake (Table 2). Each 0.1 unit increase in classroom fidelity was associated with decreased intake of 100% fruit juice (0.95; Cl: 0.91–0.99) over time. Higher high-traffic water station fidelity was associated with decreased intake of SSBs (0.94; Cl: 0.91–0.98) and plain milk (0.92; Cl: 0.89–0.96) over time. Higher cafeteria fidelity score was associated with a decrease in mean BMI z-score over time (0.027; Cl: −0.046, −0.008). No significant associations were observed for home-engagement activity completion.

**Table 2:** Mixed-effects linear regression models^1^ assessing the association of *Water First *intervention implementation with students’ beverage intake and body mass index z-score

Outcome	Time*Classroom Fidelity Score^4^			Time*High-Traffic Fidelity Score^5^			Time*Cafeteria Fidelity Score^6^			Time*Home-Engagement Score^7^		
	Est.	CI	*P*	Est.	CI	*P*	Est.	CI	*P*	Est.	CI	*P*
Intake of water^2^	0.99	0.95, 1.02	0.48	0.98	0.95, 1.01	0.13	0.98	0.92, 1.05	0.62	0.99	0.98, 1.01	0.35
Intake of SSB^2^	0.97	0.93, 1.01	0.13	0.94	0.91, 0.98	<0.001	0.98	0.91, 1.06	0.63	1.00	0.99, 1.01	0.92
Intake of plain milk^2^	1.03	0.98, 1.07	0.23	0.92	0.89, 0.96	<0.001	0.96	0.88, 1.05	0.37	0.99	0.97, 1.00	0.11
Intake of flavored milk^2^	0.97	0.93, 1.01	0.16	0.99	0.96, 1.03	0.58	1.04	0.96, 1.14	0.32	1.00	0.99, 1.01	0.98
Intake of 100% juice^2^	0.95	0.91, 0.99	0.02	0.97	0.93, 1.00	0.07	1.01	0.93, 1.11	0.77	1.00	0.99, 1.02	0.52
BMI z-score^3^	0.004	-0.006, 0.014	0.42	-0.001	-0.009, 0.007	0.76	-0.027	-0.046, -0.008	0.006	-0.002	-0.006, 0.001	0.15

*Abbreviations*: *SSB* Sugar-sweetened beverages, *BMI* Body mass index, *CI* Confidence interval

^1^Mixed-effects linear regression models adjusted for students’ ethnicity, gender, and age with random intercepts for student, school, and classroom. The primary predictor was the fidelity score (0.1 unit increase) by time interaction

^2^Beverage intake frequencies were log-transformed, and regression coefficients were exponentiated to derive the percent change in each outcome. Values greater than 1 suggest an increase, while those less than 1 suggest a decrease in beverage intake frequencies

^3^BMI z-score values were not log-transformed. Values greater than 0 suggest an increase, while those less than 0 suggest a decrease in BMI z-score

^4^Classroom fidelity score includes visibility of water promotional material (emoji decals, posters), study-provided water bottles on student desks, and teacher water bottle, and appeal of classroom water source

^5^High-traffic fidelity score includes visibility of water promotional material (emoji decals, posters) and appeal of water station

^6^Cafeteria fidelity score includes visibility of water promotional material (emoji decals, posters), availability of water dispenser and cups, and appeal of cafeteria water source

^7^Home-engagement score was calculated as the percent of *Water First* take-home activities completed per student

## Discussion

We evaluated implementation of the *Water First* water promotion and access intervention in low-income elementary schools in California’s San Francisco Bay Area. Although documenting implementation fidelity is critical for translating interventions into wider practice and policy [20, 26], this was the first analysis of fidelity for a water promotion intervention in a U.S. school setting. We found that the *Water First* intervention was largely implemented with high fidelity for school components, with mean fidelity scores ranging from 0.66 at high-traffic school water stations to 0.87 in the cafeteria. Implementation fidelity was not associated with frequency of water intake among children exposed to *Water First*. However, various components of the intervention were associated with decreases in intake of other beverages and BMI z-score. The lowest implementation was seen with the home-engagement activities, with students completing on average 41% of activities, and completion of these activities was not associated with changes in beverage intake or BMI z-score.

On the whole, most elements of the *Water First* intervention were implemented with high fidelity, with prior research noting that positive results have often been obtained with fidelity levels around 60%, with few studies attaining levels greater than 80% [27]. This suggests that teachers, cafeteria staff, and other school personnel were able to adequately implement various components of the intervention. In contrast, a kindergarten-based intervention focusing on water intake and beverage consumption in six European countries reported poor implementation scores (< 60%) [22]. However, this intervention required teachers to not only institute environmental changes in the classroom, but also implement classroom activities (stories, games, excursions, etc.), and thus may have been more burdensome for school staff than *Water First*. Notably, this European study found no significant effects of the intervention on water consumption.

The *Water First* study found that students exposed to the intervention had a significantly greater increase in the frequency of water consumed compared to control students at 7-months, an effect that was sustained at 15-months [8]. However, in the present analysis, we found no statistically significant associations between the fidelity of implementing *Water First* and student water intake. Because the major focus of the *Water First* intervention was on increasing water intake, incremental improvements in fidelity may not have translated into changes in intake. In other words, a ceiling effect may have been achieved whereby the intervention was enough to impact water intake regardless of small amounts of variation in the fidelity of its implementation. Changes to the physical school environment and social norms resulting from water promotion activities may, for example, have contributed to increased water consumption.

We found that implementation fidelity in the cafeteria was high for both the environment (mean: 0.88) and promotion (mean: 0.84) components of the intervention, suggesting that cafeteria staff successfully provided a water dispenser with cups during lunch time and displayed study-provided water promotional materials. A prior trial in Boston found that the provision of cups in cafeterias was associated with double the intake of water from drinking fountains, suggesting that this may be a high-value component of the intervention [15]. In high-traffic areas, newly installed water stations were well-maintained (environment fidelity score mean: 0.91); however, schools struggled to maintain posters and other promotional materials near the water stations (promotion score mean: 0.54).

The fact that implementation fidelity was higher in components implemented in classrooms and cafeterias relative to high-traffic water stations is noteworthy. In classrooms and cafeterias, there are dedicated school staff responsible for maintaining the space each day, namely teachers and cafeteria staff, which may not be true for water stations in high-traffic areas. Additionally, many of the water stations in high-traffic areas were in outdoor settings. This may have led to posters and promotional materials becoming wet from rain or blowing off due to the wind. Likewise, water stations in these settings may have required more maintenance. While installation indoors would have avoided these concerns, the water stations would not be as accessible to students.

The observed decreases in SSB and 100% fruit juice consumption with higher classroom and high-traffic area water station fidelity were consistent with the goals of the intervention [17]. Messaging around the benefits of water vs. other beverages may have encouraged students to avoid drinking SSBs and 100% fruit juice. We may have seen effects on these outcomes compared to water intake because these secondary outcomes may be more responsive to small improvements in implementation fidelity. Additionally, the impact of the intervention messaging in school may spill over to the home environment wherein students are drinking less SSBs and 100% fruit juice at home.

The observed decrease in plain milk consumption with higher implementation fidelity at high-traffic water stations was an unintended consequence of the intervention, as the intervention did not encourage decreased overall milk intake. Future efforts to implement *Water First* should provide messaging to ensure that students receive adequate milk-associated nutrient intake. For example, the intervention can provide guidance about the importance of vitamin D and calcium if milk intake is reduced and offer examples of foods/beverages that are good sources of these nutrients. In addition to the observed decreases in SSB, 100% juice, and plain milk intake, we also found that higher fidelity for cafeteria components of the intervention was associated with lower BMI z-score at 15-months. This may have resulted from cumulative effects of reduced consumption of calorie-containing beverages over time. In sensitivity analyses, this change in BMI was observed between 7-months and 15-months, but not between baseline and 7-months, which is consistent with the longer time course of weight changes compared to changes in beverage intake.

Compared to other similar interventions [12, 14–16], the *Water First* intervention uniquely included a home-based component in the form of four take-home activities for students to complete with their families. Relative to other components of the intervention, average completion of these activities was low (41%) and no significant effects were observed with higher completion. Several reasons may explain our null findings. First, it is unclear to what extent students shared the activities with their families as intended vs. completed them on their own. Given the effort required to distribute, collect, and mark student homework assignments, this component of the intervention may be less beneficial for future implementation. However, engagement with parents is likely still beneficial. For example, a process evaluation of the European kindergarten-based beverage intake intervention found greater decreases in consumption of SSBs and fruit juice among children with a high parental implementation score (i.e., engagement with newsletters, tip-cards, and posters) [22]. Future efforts should explore ways of directly engaging parents (e.g., back-to-school nights), rather than relying on students to take activities home to their families. A systematic review of parental support interventions targeting children’s health behaviors found that sending information home was generally ineffective, whereas more active forms of engagement —such as parental counseling and group-based sessions—improved outcomes [28].

There are several limitations of this study relevant to the interpretation of our results. One key limitation may be inter-rater differences in evaluating implementation fidelity. Though all raters used a standardized form and followed consistent protocols, inter-rater reliability was not assessed. Additionally, this analysis focused only on objective researcher observations of the extent to which specific components of the intervention were implemented with fidelity. However, given that the intervention was conducted in the school setting, gaining a better understanding of school staff perspectives through interviews or focus groups may offer insight into how such interventions can be most feasibly and sustainably implemented long-term. Still, strengths of this study include our relatively large sample size and inclusion of comprehensive data regarding fidelity for each key component of the intervention.

## Conclusions

We found that the *Water First* intervention was largely implemented with high fidelity. Higher fidelity was not associated with increases in water intake, which may be attributable to a ceiling effect whereby the presence of the intervention was enough to impact water intake regardless of small amounts of variation in the fidelity of its implementation. Still, *Water First* was associated with decreases in SSB and 100% fruit juice intake and lower BMI z-score. Additionally, take-home activity completion was relatively low among students, with no effects on beverage intake or BMI z-score. This study adds to the very limited data evaluating fidelity for school-based water promotion interventions and may inform future implementation efforts.

## Supplementary Information

Supplementary Material 1.

Supplementary Material 2.

## Data Availability

Availability of data and materials: As data from the *Water First* study are still be used for other funded analyses, the data is currently not publicly available. Please contact the corresponding author (Anisha I. Patel, anipatel@stanford.edu) if you have questions.

## Abbreviations

US: United States
SSB: Sugar-sweetened beverage
BMI: Body mass index
NHANES: National Health and Nutrition Examination Survey
