# Supplementary material for: Fidelity evaluation of the Water First intervention for promoting tap water consumption in school-age children
Source: BMC Public Health. 2026 Jun 13;26:2399. doi: 10.1186/s12889-026-28101-w (PMC13491641; doi:10.1186/s12889-026-28101-w)
Supplement: Supplementary file 1 [file 12889_2026_28101_MOESM1_ESM.docx]

**Supplemental Materials**

| Supplemental Table 1. Student demographic characteristics and study outcomes for the intervention arm (N = 9 schools) of the *Water First* Study. | | |
| --- | --- | --- |
| **Variable** | **No. Students** | **Mean (SD)** |
| **Demographics** |  |  |
| Age | 677 | 9.63 (0.44) |
| Female Gender (N, %) | 677 | 318 (47.0) |
| Hispanic Ethnicity (N, %) | 675 | 492 (72.9) |
| **Outcomes** |  |  |
| Intake of water, times/day - baseline | 677 | 6.06 (4.30) |
| Intake of water, times/day – 7 months | 639 | 7.10 (5.14) |
| Intake of water, times/day – 15 months | 548 | 6.25 (4.71) |
| Intake of SSB, times/day – baseline | 677 | 2.48 (2.76) |
| Intake of SSB, times/day – 7 months | 639 | 1.83 (2.36) |
| Intake of SSB, times/day – 15 months | 548 | 1.57 (1.84) |
| Intake of plain milk, times/day – baseline | 677 | 1.17 (1.35) |
| Intake of plain milk, times/day – 7 months | 639 | 1.17 (1.57) |
| Intake of plain milk, times/day – 15 months | 548 | 1.08 (1.36) |
| Intake of flavored milk, times/day – baseline | 677 | 0.57 (0.94) |
| Intake of flavored milk, times/day – 7 months | 639 | 0.37 (0.69) |
| Intake of flavored milk, times/day – 15 months | 548 | 0.40 (0.78) |
| Intake of 100% juice, times/day – baseline | 677 | 0.79 (1.12) |
| Intake of 100% juice, times/day – 7 months | 639 | 0.63 (1.03) |
| Intake of 100% juice, times/day – 15 months | 548 | 0.54 (1.02) |
| BMI z-score – baseline | 677 | 0.91 (1.13) |
| BMI z-score – 7 months | 640 | 0.93 (1.10) |
| BMI z-score – 15 months | 548 | 0.93 (1.14) |

| Supplemental Table 2. Fidelity scores by location. | | |
| --- | --- | --- |
|  | No. Units | Mean (SD) |
| Classroom Fidelity Score – Overall | 28 classrooms | 0.73 (0.13) |
| Classroom Fidelity Score – Promotion | 28 classrooms | 0.80 (0.16) |
| Classroom Fidelity Score – Environment | 28 classrooms | 0.69 (0.14) |
| High-Traffic Fidelity Score – Overall | 9 schools | 0.66 (0.15) |
| High-Traffic Fidelity Score – Promotion | 9 schools | 0.54 (0.22) |
| High-Traffic Fidelity Score – Environment | 9 schools | 0.91 (0.06) |
| Cafeteria Fidelity Score – Overall | 9 schools | 0.87 (0.06) |
| Cafeteria Fidelity Score – Promotion | 9 schools | 0.84 (0.13) |
| Cafeteria Fidelity Score – Environment | 9 schools | 0.88 (0.06) |
| Home-Engagement Score | 664 | 0.41 (0.33) |

| Supplemental Table 3a. Mixed-effects linear regression models^1^ assessing the association of *Water First* intervention implementation with students’ beverage intake and body mass index z-score, baseline to 7-months. | | | | | | | | | | | | | | | |
| --- | --- | --- | --- | --- | --- | --- | --- | --- | --- | --- | --- | --- | --- | --- | --- |
| Outcome | Time*Classroom Fidelity Score^4^ | | | | Time*High-Traffic Fidelity Score^5^ | | | | Time*Cafeteria Fidelity Score^6^ | | | Time*Home-Engagement Score^7^ | | | |
|  | Est. | CI | *P* | Est. | | CI | *P* | Est. | | CI | *P* | Est. | CI | *P* |  |
| Intake of water^2^ | 1.03 | 0.97, 1.09 | 0.35 | 1.00 | | 0.95, 1.06 | 0.87 | 1.05 | | 0.92, 1.20 | 0.47 | 0.99 | 0.96, 1.01 | 0.25 |  |
| Intake of SSB^2^ | 0.93 | 0.87, 1.00 | 0.04 | 0.96 | | 0.90, 1.02 | 0.15 | 1.15 | | 0.99, 1.34 | 0.07 | 1.00 | 0.97, 1.03 | 0.99 |  |
| Intake of plain milk^2^ | 0.99 | 0.92, 1.07 | 0.78 | 0.88 | | 0.82, 0.95 | <0.001 | 0.96 | | 0.81, 1.15 | 0.69 | 0.98 | 0.95, 1.01 | 0.17 |  |
| Intake of flavored milk^2^ | 0.95 | 0.88, 1.02 | 0.17 | 1.00 | | 0.94, 1.07 | 0.94 | 1.06 | | 0.90, 1.25 | 0.48 | 1.00 | 0.97, 1.03 | 0.87 |  |
| Intake of 100% juice^2^ | 0.95 | 0.88, 1.02 | 0.16 | 0.94 | | 0.88, 1.01 | 0.09 | 1.03 | | 0.87, 1.22 | 0.75 | 1.01 | 0.98, 1.04 | 0.50 |  |
| BMI z-score^3^ | 0.0004 | -0.016, 0.017 | 0.96 | -0.005 | | -0.021, 0.01 | 0.49 | 0.0002 | | -0.036, 0.037 | 0.99 | -0.001 | -0.007, 0.005 | 0.76 |  |
| ^1^Mixed-effects linear regression models adjusted for students’ ethnicity, gender, and age with random intercepts for student, school, and classroom. The primary predictor was the fidelity score (0.1 unit increase) by time interaction.  ^2^Beverage intake frequencies were log-transformed, and regression coefficients were exponentiated to derive the percent change in each outcome. Values greater than 1 suggest an increase, while those less than 1 suggest a decrease in beverage frequencies.  ^3^BMI z-score values were not log-transformed. Values greater than 0 suggest an increase, while those less than 0 suggest a decrease in BMI z-score.  ^4^Classroom fidelity score includes visibility of water promotional material (emoji decals, posters), study-provided water bottles on student desks, and teacher water bottle, and appeal of classroom water source.  ^5^High-traffic fidelity score includes visibility of water promotional material (emoji decals, posters) and appeal of water station.  ^6^Cafeteria fidelity score includes visibility of water promotional material (emoji decals, posters), availability of water dispenser and cups, and appeal of cafeteria water source.  ^7^Home-engagement score was calculated as the percent of *Water First* take-home activities completed per student.  Abbreviations: sugar-sweetened beverages (SSB), body mass index (BMI), confidence interval (CI). | | | | | | | | | | | | | | | |

| Supplemental Table 3b. Mixed-effects linear regression models^1^ assessing the association of *Water First* intervention implementation with students’ beverage intake and body mass index z-score, 7-months to 15-months. | | | | | | | | | | | | | | | |
| --- | --- | --- | --- | --- | --- | --- | --- | --- | --- | --- | --- | --- | --- | --- | --- |
| Outcome | Time*Classroom Fidelity Score^4^ | | | | Time*High-Traffic Fidelity Score^5^ | | | | Time*Cafeteria Fidelity Score^6^ | | | Time*Home-Engagement Score^7^ | | |  |
|  | Est. | CI | *P* | Est. | | CI | *P* | Est. | | CI | *P* | Est. | CI | *P* |  |
| Intake of water^2^ | 0.95 | 0.89, 1.02 | 0.15 | 0.95 | | 0.90, 1.01 | 0.11 | 0.92 | | 0.80, 1.06 | 0.23 | 1 | 0.98, 1.03 | 0.79 |  |
| Intake of SSB^2^ | 1.02 | 0.94, 1.10 | 0.68 | 0.93 | | 0.87, 0.99 | 0.03 | 0.83 | | 0.71, 0.97 | 0.02 | 1 | 0.97, 1.03 | 0.92 |  |
| Intake of plain milk^2^ | 1.08 | 0.98, 1.18 | 0.11 | 0.97 | | 0.90, 1.05 | 0.47 | 0.95 | | 0.79, 1.15 | 0.61 | 1 | 0.97, 1.03 | 0.81 |  |
| Intake of flavored milk^2^ | 0.99 | 0.91, 1.07 | 0.75 | 0.97 | | 0.91, 1.04 | 0.45 | 1.02 | | 0.86, 1.22 | 0.78 | 1 | 0.97, 1.03 | 0.81 |  |
| Intake of 100% juice^2^ | 0.95 | 0.87, 1.04 | 0.24 | 0.99 | | 0.92, 1.07 | 0.87 | 1.00 | | 0.83, 1.19 | 0.97 | 1 | 0.97, 1.03 | 0.97 |  |
| BMI z-score^3^ | 0.008 | -0.011, 0.027 | 0.41 | 0.003 | | -0.013, 0.019 | 0.70 | -0.056 | | -0.094, -0.017 | 0.004 | -0.004 | -0.011, 0.003 | 0.24 |  |
| ^1^Mixed-effects linear regression models adjusted for students’ ethnicity, gender, and age with random intercepts for student, school, and classroom. The primary predictor was the fidelity score (0.1 unit increase) by time interaction.  ^2^Beverage intake frequencies were log-transformed, and regression coefficients were exponentiated to derive the percent change in each outcome. Values greater than 1 suggest an increase, while those less than 1 suggest a decrease in beverage frequencies.  ^3^BMI z-score values were not log-transformed. Values greater than 0 suggest an increase, while those less than 0 suggest a decrease in BMI z-score.  ^4^Classroom fidelity score includes visibility of water promotional material (emoji decals, posters), study-provided water bottles on student desks, and teacher water bottle, and appeal of classroom water source.  ^5^High-traffic fidelity score includes visibility of water promotional material (emoji decals, posters) and appeal of water station.  ^6^Cafeteria fidelity score includes visibility of water promotional material (emoji decals, posters), availability of water dispenser and cups, and appeal of cafeteria water source.  ^7^Home-engagement score was calculated as the percent of *Water First* take-home activities completed per student.  Abbreviations: sugar-sweetened beverages (SSB), body mass index (BMI), confidence interval (CI). | | | | | | | | | | | | | | | |

**Supplemental Figures.** Examples of promotional materials used in the *Water First* intervention. Additional resources are available here: <https://researchinchildhealth.org/resources/water-first>.
